# Inter-laboratory analysis of selected genetically modified plant reference materials with digital PCR

**DOI:** 10.1007/s00216-017-0711-1

**Published:** 2017-10-25

**Authors:** David Dobnik, Tina Demšar, Ingrid Huber, Lars Gerdes, Sylvia Broeders, Nancy Roosens, Frederic Debode, Gilbert Berben, Jana Žel

**Affiliations:** 1 Department of Biotechnology and Systems Biology, National Institute of Biology (NIB), Večna pot 111, 1000 Ljubljana, Slovenia; 2 Bavarian Health and Food Safety Authority (LGL), Veterinärstraße 2, 85764 Oberschleißheim, Germany; 3 Scientific Institute of Public Health (WIV-ISP), J. Wytsmanstraat 14, 1050 Brussels, Belgium; 4 Walloon Agricultural Research Centre (CRA-W), Chaussée de Namur 24, 5030 Gembloux, Belgium

**Keywords:** Digital PCR, Droplet digital PCR, Absolute quantification, Reference materials, GMO quantification

## Abstract

Digital PCR (dPCR), as a new technology in the field of genetically modified (GM) organism (GMO) testing, enables determination of absolute target copy numbers. The purpose of our study was to test the transferability of methods designed for quantitative PCR (qPCR) to dPCR and to carry out an inter-laboratory comparison of the performance of two different dPCR platforms when determining the absolute GM copy numbers and GM copy number ratio in reference materials certified for GM content in mass fraction. Overall results in terms of measured GM% were within acceptable variation limits for both tested dPCR systems. However, the determined absolute copy numbers for individual genes or events showed higher variability between laboratories in one third of the cases, most possibly due to variability in the technical work, droplet size variability, and analysis of the raw data. GMO quantification with dPCR and qPCR was comparable. As methods originally designed for qPCR performed well in dPCR systems, already validated qPCR assays can most generally be used for dPCR technology with the purpose of GMO detection.

**Graphical abstract:**
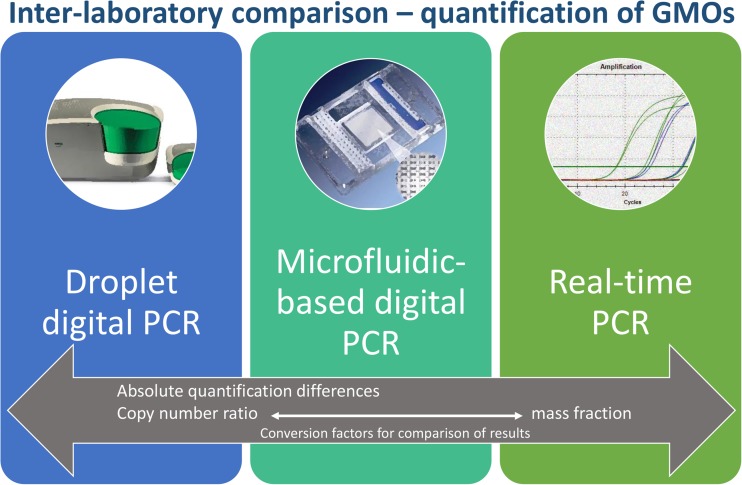
The output of three different PCR-based platforms was assessed in an inter-laboratory comparison

The output of three different PCR-based platforms was assessed in an inter-laboratory comparison

## Introduction

Accurate and comparable measurements in the field of genetically modified (GM) organisms (GMOs) detection are achieved by using validated methods and appropriate calibrators as an anchor point for the measurement value and measurement units [1]. In the European Union, according to Commission Regulation (EU Commission (EC)) No. 641/2004 [2], reference materials (RMs) with a certified content of the specific targets are needed for the calibration of quantitative real-time PCR (qPCR) measurements. RMs are available in different types, including whole seeds, seed-derived powder, genomic DNA (gDNA) from leaves, and plasmids. The majority of commercially available RMs are certified for GM content in mass fraction, whereas only few are certified for copy number ratios [3, 4].

Quantification result of GMOs with qPCR is mostly expressed as mass fraction; however, with the emergence of new techniques, such as digital PCR (dPCR), the copy number ratio might be used more frequently. Digital PCR enables precise quantification of DNA targets. As it is determining the absolute number of copies in the sample, the final result of a GM quantification is given as a copy number ratio of the GM event and taxon-specific gene. It is important to note that due to different units of measurement, the data from different quantification procedures for GMOs must be converted to be comparable. Out of several regulations from the EC, none covers the unit of measurement for all of the possible cases, but they rather address specific topics (e.g., Regulation (EC) 619/2011 [5]). Thus, in the field of GMO detection and quantification, it is an ongoing challenge to define the relation between the transgene/endogen ratios expressed as mass fraction and as copy number.

Nowadays, the copy numbers expressed as haploid genome equivalents (HGE) are calculated on the basis of the measured concentration of the isolated genomic DNA, the zygosity, and the size of the plant genome [6]. These values are good approximations of the real copy number value but, unfortunately, these data are not available for all RMs. As these materials are also used for method validation, calculated copy number values could introduce an error, influencing the reported performance of the method. An error in the estimation of copy numbers could result in non-compliance with required minimum performance parameters, if the actual amount of copies present would be, for example, higher than calculated. Thus, employing absolute quantification with dPCR could be a solution to get the most accurate copy number value.

In view of different regulations and measurement techniques, a guidance document was prepared by EC Joint Research Centre (JRC) [7], which recommends the use of a conversion factor from copy number into mass fraction. As proposed in this guidance document [7], a special study should be conducted to determine conversion factors with associated uncertainty for RMs used in GMO quantification. Studies on suitability of dPCR for GMO quantification were already reported [8–14]; however, none of the studies included the inter-laboratory comparison of two dPCR platforms on a high number of RMs. The purpose of our study was multiple. Firstly, the aim was to test the transferability of a wide range of GMO quantification methods, designed for qPCR, to dPCR. Secondly, an inter-laboratory comparison of the performance of two different dPCR platforms when determining the absolute GM copy numbers and GM content by using plant materials (including RMs certified for their GM content) was carried out. An additional goal was to evaluate the accuracy of copy number calculation based on DNA concentration and genome mass, by testing different materials used for in-house validation of the methods. The inter-laboratory studies reported in this manuscript were undertaken in the frame of the GMOval project [15]. We have shown that GM content determination is comparable between laboratories and platforms; however, determination of absolute copy number values was more biased between the laboratories. With this study, we have made a first contribution towards reliable determination of RM-specific factors that enable conversion between the copy number and mass fraction values.

## Materials and methods

### Test materials

Reference materials certified for their GM content were purchased from the Joint Research Centre Geel (JRC Geel, Belgium), formerly Institute for Reference Materials and Measurements (IRMM, Geel, Belgium), and the American Oil Chemists’ Society (AOCS, Urbana, IL, USA) as powders, except for T25, A2704, and LL62, which were in the form of leaf genomic DNA. Commercial pea seeds were obtained from the Vilmorin Company (France). Two real-life samples, maize flour (G154/13) and biscuits (G035/14), were selected out of routine analyses samples for official GMO detection at the National Institute of Biology (NIB). Experiments were performed in four different laboratories using the same DNA material.

### DNA extraction

Genomic DNA from all plant materials and the real-life samples was extracted using the CTAB method (ISO21571 [16] with minor modifications), except for Bt176 and Bt11, where the Nucleospin Food Kit (Macherey-Nagel, Düren, Germany) was used. The sample intake was 200 mg or 1 g per isolation for the CTAB extractions and 200 mg per isolation for the Nucleospin Food Kit. Quantity of DNA used in the reactions described below was from 100 to 10 ng per reaction.

### Digital droplet PCR

Each DNA sample (i.e., CRM, pea, real-life sample) was tested in two dilutions (the actual dilutions were different between materials and were determined based on the quantity of isolated DNA [data not shown]), both in duplicate for endogene, event, and/or screening element (simplex reactions). The endogenes for different species were hmgA for maize, lectin (Lec) for soybean, pea lectin for pea, PLD for rice, and PepC for oilseed rape.

Primer and probe concentrations for GM events were as in the European Union Reference Laboratory (EURL) validated methods [17] or as reported by Debode et al. (2017) [18] for screening elements (tE9, pat, bar) and pea lectin. For the Bt176 event, the method described in Brodmann et al. (2002) [19] was used. For hmgA and PepC, primers and probes were not from the abovementioned validated methods and were as follows: HMG-F 5′-TTGGACTAGAAATCTCGTGCTGA-3′ (final concentration 300 nM), HMG-R 5′-GCTACATAGGGAGCCTTGTCCT-3′ (final concentration 300 nM), HMG-P FAM-5′-CAATCCACACAAACGCACGCGTA-3′-BHQ (final concentration 180 nM), PEP-F 5′-CAGTTCTTGGAGCCGCTTGAG-3′ (final concentration 900 nM), PEP-R 5′-TGACGGATGTCGAGCTTCACA-3′ (final concentration 900 nM), and PEP-P 6-FAM-ACAGACCTACAGCCGATGGAAGCCTGC-TAMRA (final concentration 200 nM).

Twenty microliters of sample mix was loaded in the well of the cartridge for droplet generation (10 μl of Master Mix (2× digital droplet PCR (ddPCR) Supermix for probes, Bio-Rad, Pleasanton, CA), 6 μl of primers and probes mix, 4 μl of DNA). After droplets were generated, they were transferred to a 96-well plate, sealed, and inserted into a T100 Thermal cycler (Bio-Rad, Pleasanton, CA). The following cycling program was used: 2 min at 50 °C, 10 min at 95 °C, 40 cycles of denaturation and annealing (15 s at 95 °C and 1 min at 60 °C), and final denaturation at 98 °C followed by cooling to 4 °C. After PCR cycling, the plate was inserted into a droplet reader machine (QX100 droplet reader, Bio-Rad, Pleasanton, CA), where the fluorescence of droplets was analyzed. Data acquisition and analysis were performed using the QuantaSoft (Bio-Rad, Pleasanton, CA) software. Positive droplets, containing amplification products, were discriminated from negative droplets without amplification products by applying a fluorescence amplitude threshold. The threshold was set manually, using both the fluorescence amplitude vs. event number and the histogram of events vs. amplitude data streams, on each of the FAM or VIC/HEX channels. Data generated by the QX100 droplet reader were rejected from subsequent analysis if a low number (< 8000) of droplets was measured per 20 μL reaction. After being exported, the data were further analyzed using Microsoft Excel spreadsheets. Results of estimated target concentration per reaction were used for the calculation of the initial number of target copies present in the stock material. Coefficient of variation (cv) was calculated from eight or four replicates, if it was calculated for average of both labs or individual lab, respectively. The experiments were performed in two independent laboratories (NIB and Bavarian Health and Food Safety Authority).

### Microfluidic-based digital PCR

Each DNA sample was tested in one dilution, but two technical replicates were made twice (qPCR mix prepared for four replicates and divided in two aliquots, then DNA was added to both aliquots separately and two inlets were filled from each of the two aliquots). The final volume per inlet was 4 μl (2 μl TaqMan Master Mix, 0.4 μl 20× GE Sample Loading reagent, 0.4 μl of primers and probes mix, and 1.2 μl DNA). Primer and probe concentrations were as in ddPCR reactions.

The reaction mix was loaded on the Fluidigm dPCR 37K IFC chip, inserted into BioMark HD machine (Fluidigm, Markham, Canada), and run with the following program: 2 min at 50 °C, 10 min at 95 °C, and 45 cycles of denaturation and annealing (15 s at 95 °C and 1 min at 60 °C).

Results of estimated targets per panel were used for the calculation of the initial number of target copies present in the stock material. CV was calculated from eight or four replicates, if it was calculated for average of both labs or individual lab, respectively. The experiments were performed in two independent laboratories (NIB and Walloon Agricultural Research Centre).

### Quantitative real-time PC

The content of five transgenic events in the two real-life samples was determined by qPCR, using relative quantification according to the standard curve approach. Standard curves were prepared by making serial dilutions (five steps) of the respective CRM (starting from approximately 100 to 1 ng DNA per reaction) and used in two or three replicates. For each sample, the quantification was done based on two replicates of two dilutions. Results of quantification performed with CRM certified for transgene mass fraction were also expressed in this way. The experiments were performed in two independent laboratories (NIB and Scientific Institute of Public Health).

## Results

### Inter-laboratory determination of copy numbers with ddPCR

For each material, the absolute copy number per microliter was determined for the endogene and event, and the average was calculated for all results together and per laboratory (Table 1). For the five materials used in the GMOval project validations (pea, GT73, MON88017, Bt176, and Bt11), copy number concentrations of the respective screening elements were determined additionally (except for Cry3Bb in MON88017).

**Table 1:** Absolute copy numbers determined by ddPCR in two laboratories

DNA sample	Target	Average copies/μl	cv %	Average copies/μl laboratory 1	Average copies/μl laboratory 2	% bias laboratory 2 to laboratory 1
Pea	LecPea	181,703	8.38	195,268	168,138	− 13.9
	tE9	135,106	3.10	136,168	134,044	− 1.56
GT73	PepC	458,581	7.76	491,325	425,837	− 13.3
	tE9	349,261	3.67	356,226	342,295	− 3.91
	GT73	258,219	6.38	270,428	246,010	− 9.03
MON88017	hmgA	79,646	5.12	83,281	76,011	− 8.73
	MON88017	42,373	5.53	44,370	40,375	− 9.00
Bt176	hmgA	97,300	0.73	97,300	97,300	0.00
	Bt176	4416	18.2	3668	5164	**40.8**
	bar	4251	7.47	4431	4070	− 8.14
Bt11	hmgA	98,250	4.87	102,350	94,150	− 8.01
	Bt11	1685	6.67	1714	1655	− 3.41
	pat	1570	6.08	1612	1528	− 5.19
MON40-3-2	Lec	104,435	22.0	83,466	125,403	**50.2**
	MON40-3-2	11,070	18.0	9243	12,896	**39.5**
MON89788	Lec	33,148	12.4	29,339	36,957	**26.0**
	MON89788	31,139	12.7	27,480	34,798	**26.6**
DP98140	hmgA	36,258	7.58	33,896	38,621	13.9
	DP98140	2870	5.17	2759	2982	8.09
MON863	hmgA	28,365	10.0	25,733	30,997	20.5
	MON863	1723	11.2	1545	1901	23.0
MON810 × MON863	hmgA	72,199	2.75	73,806	70,593	− 4.35
	MON863	3628	**25.7**	4497	2760	**− 38.6**
	MON810	3687	15.5	3163	4212	**33.2**
DAS59122	hmgA	89,977	24.9	69,106	110,849	**60.4**
	DAS59122	3121	24.3	2437	3805	**56.2**
DAS1507	hmgA	25,240	7.86	23,546	26,934	14.4
	DAS1507	1441	8.77	1342	1540	14.7
NK603	hmgA	28,036	17.4	23,506	32,566	**38.5**
	NK603	694	17.4	594	794	**33.7**
MIR162	hmgA	179,381	19.6	147,109	211,653	**43.9**
	MIR162	105,160	17.0	88,960	121,360	**36.4**
MIR604	hmgA	45,439	16.3	38,684	52,194	**34.9**
	MIR604	17,288	9.78	15,796	18,780	18.9
GA21	hmgA	52,260	7.49	49,909	54,610	9.42
	GA21	17,689	4.97	17,804	17,574	− 1.29
T25	hmgA	400,942	4.88	403,428	398,455	− 1.23
	T25	386,050	5.03	385,245	386,855	0.42
MON89034	hmgA	68,802	14.1	59,889	77,715	**29.8**
	MON89034	41,582	16.9	35,085	48,078	**37.0**
MON87460	hmgA	116,865	6.5	110,610	123,119	11.3
	MON87460	45,024	3.05	45,053	44,995	− 0.13
MON810	hmgA	95,927	21.0	77,257	114,597	**48.3**
	MON810	3276	24.8	2656	3896	**46.7**
LL62	PLD	245,909	1.81	243,518	248,300	1.96
	LL62	183,605	4.09	178,888	188,322	5.27
A2704	Lec	454,412	**35.4**	304,457	604,366	**98.5**
	A2704	452,472	**31.6**	319,376	585,568	**83.4**
G035/14	Lec	12,180	3.38	12,066	12,408	2.84
	MON40-3-2	5966	3.95	5854	6077	3.81
	MON89788	2580	5.58	2467	2692	9.13
G154/13	hmgA	33,199	2.57	32,768	33,630	2.63
	59122	358	7.42	352	363	3.16
	TC1507	757	9.33	707	806	14.1
	NK603	681	8.11	651	711	9.08

Absolute bias values of > 25% are shown in bold

The results of average absolute copy numbers determined over the two laboratories for each event or gene had the overall cv higher than 25% (below this threshold variability is acceptable for GMO methods [20]) in only 3 out of 55 cases (Table 1). Further, when comparing the average values determined individually between both laboratories, there were 20 out of 55 cases where the absolute bias was over 25% (Table 1).

Based on the absolute copy numbers measured with ddPCR, the GM% for all materials was calculated (Table 2). For all of the tested screening elements and events, the cv was lower than 25%, when combining results of both laboratories. When comparing the results for each screening element and event between both laboratories, a bias higher than the accepted value (25%) was observed only for the Bt176 event (Table 2). For other events or genes, the absolute bias did not exceed 25%. No tendency for one laboratory to produce over- or under-estimated results was observed, since the calculated bias was deviating in both directions (Table 2).

**Table 2:** GM% in samples calculated based on the determined copy numbers

DNA sample	Target	Average GM%	cv %	Average GM% laboratory 1	Average GM% laboratory 2	% bias laboratory 2 to laboratory 1
Pea	tE9	74.8^a^	10	69.8	79.9	14.6
GT73	tE9	76.4	6.31	72.5	80.4	10.8
	GT73	56.4	5.58	55	57.8	5.02
MON88017	MON88017	53.2	2.29	53.3	53.1	− 0.30
Bt176	Bt176	4.43	20.48	3.66	5.2	**42.3**
	bar	4.26	5.12	4.42	4.1	− 7.19
Bt11	Bt11	1.73	3.85	1.72	1.73	0.77
	pat	1.61	7.67	1.62	1.6	− 1.50
MON40-3-2	MON40-3-2	10.7	7.03	11.1	10.3	− 7.15
MON89788	MON89788	93.9	2.65	93.7	94.2	0.48
DP98140	DP98140	7.94	6.01	8.15	7.73	− 5.19
MON863	MON863	6.07	3.01	6	6.14	2.18
MON810 × MON863	MON863	6.03	1.25	6.09	5.97	− 2.08
	MON810	4.10	6.35	4.29	3.91	− 8.80
DAS59122	DAS59122	3.48	1.73	3.53	3.43	− 2.65
DAS1507	DAS1507	5.71	2.32	5.7	5.72	0.25
NK603	NK603	2.48	2.91	2.53	2.44	− 3.51
MIR162	MIR162	59.0	6.98	60.5	57.5	− 4.93
MIR604	MIR604	38.5	9.61	40.9	36	− 11.8
GA21	GA21	34.1	12	35.7	32.5	− 8.99
T25	T25	96.4	6.34	95.7	97.2	1.63
MON89034	MON89034	60.3	5.69	58.6	62	5.77
MON87460	MON87460	38.7	8.44	40.8	36.6	− 10.2
MON810	MON810	3.83	4.27	3.91	3.75	− 4.19
LL62	LL62	74.7	2	73.5	75.9	3.25
A2704	A2704	101	6.79	105	96.9	− 7.79
G035/14	MON40-3-2	48.8	5.52	48.5	49.1	1.07
	MON89788	21.1	4.71	20.5	21.7	6.08
G154/13	DAS59122	1.08	4.53	1.07	1.08	0.63
	DAS1507	2.28	7.45	2.16	2.4	11.2
	NK603	2.05	8.72	1.99	2.11	6.22

Absolute bias value of > 25% is shown in bold

^a^The value is not an actual GM% but ratio of two endogene elements with different number of alleles

The results of GM% obtained with ddPCR are reported in copy number ratio (event/endogene), whereas the certificates of the CRMs report the content of the GM event in mass fraction. In Table 3, the calculated GM% values (average over two laboratories) are presented in comparison to the certified values, together with the conversion to mass fraction according to the EURL technical guidance 619/2011 [5]. The problem of the conversion is most pronounced in the case of heterozygous maize seeds (e.g., MIR604), where the parental origin is unknown. Due to the seed composition, it is important to know if the GM parent is male or female. In case of a male GM parent, the actual GM DNA content ratio is 0.34–0.39 per seed and 0.57–0.66 in case of a female GM parent [21]. Based on the data presented in Table 3 (ratio between measured GM% and certified GM%), it can be seen that the actual measurements fall into the theoretical categories mentioned above and we can correctly deduce whether the GM parent was male or female. A nice example is the case of the MON810xMON863 stacked event, where both individual transgenic maize lines were crossed. From the data, we can see that the parents were MON863 as female and MON810 as male.

**Table 3:** Comparison between GM% determined by ddPCR and certified GM%

CRM	Certified GM% (mass fraction)	Calculated GM% (copy number ratio; average of two laboratories)	Calculated GM%/certified GM%	GM%^a^ (mass fraction)	% bias of calculated mass fraction to certified GM% in mass fraction
GT73	99.19	56.4	0.57	113	13.8
MON88017	99.05	53.2	0.54	107	7.50
Bt176	5.00	4.14	0.83	8.27	**65.4**
Bt11	4.89	1.73	0.35	3.46	**− 29.2**
MON40-3-2	10.00	10.7	1.07	10.7^b^	7.00
MON89788	99.40	93.9	0.94	93.9^b^	− 5.60
A2704	99.99	101	1.01	101^b^	0.90
LL62	99.99	74.7	0.75	74.7^b^	**− 25.3**
DP98140	10.00	7.94	0.79	15.9	**58.9**
MON863	9.85	6.07	0.62	12.1	23.2
DAS1507	9.86	5.70	0.58	11.4	15.6
NK603	4.91	2.48	0.51	4.96	1.00
DAS59122	9.87	3.48	0.35	6.96	**− 29.5**
MON810	9.90	3.83	0.39	7.66	− 22.6
MIR162	99.88	59.0	0.59	118	18.1
MIR604	99.98	38.5	0.38	76.9	− 23.1
GA21	99.98	34.1	0.34	68.2	**− 31.8**
T25	99.99	96.5	0.97	96.5^b^	− 3.40
MON89034	99.43	60.2	0.61	120	21.2
MON87460	99.05	38.7	0.39	77.3	− 21.9
MON810 x MON863 (target MON863)	9.85	6.03	0.61	12.1	22.4
MON810 × MON863 (target MON810)	9.85	4.10	0.42	8.19	− 16.9

Absolute bias values of > 25% are shown in bold

^a^Value converted from measured GM% (in copies) to mass ratio according to EURL technical guidance 619/2011 [5]

^b^Conversion factor of 1 was used as the material was homozygous

### Inter-laboratory copy number determination using microfluidic-based dPCR

The five materials used in in-house validations during the GMOval project (pea, GT73, MON88017, Bt176, and Bt11) were also analyzed with another dPCR system, a microfluidic-based dPCR. In contrast to ddPCR, the microfluidic-based dPCR has a much narrower dynamic range (~ 200 to 700 copies per reaction, compared to the 1 to 100,000 copies per reaction range in ddPCR), which has to be taken into account when preparing the dilutions of the materials/samples. Like in ddPCR, the copy numbers were measured (Table 4) and used to calculate the corresponding GM% of the materials (Table 5).

**Table 4:** Absolute copy numbers determined by microfluidic-based dPCR

DNA sample	Target	Average copies/μl	cv %	Average copies/μl laboratory 3	Average copies/μl laboratory 2	% bias laboratory 3 to laboratory 2	% bias microfluidic-based dPCR to ddPCR^a^
Pea	LecPea	228,779	9.00	213,967	243,590	13.8	7.84
	tE9	208,217	5.10	200,216	216,218	7.99	24.2
GT73	PepC	613,739	6.14	595,811	631,667	6.02	18.18
	tE9	519,057	3.99	531,704	506,410	− 4.76	25.3
	GT73	352,934	8.59	355,997	349,872	− 1.72	19.9
MON88017	hmgA	91,599	11.5	86,122	97,077	12.7	5.09
	MON88017	49,353	8.79	46,040	52,667	14.4	6.20
Bt176	hmgA	113,168	9.60	103,323	123,013	19.1	7.28
	Bt176	4822	19.4	4095	5549	**35.5**	9.42
	bar	4760	7.37	5011	4424	− 11.7	− 0.88
Bt11	hmgA	108,534	10.2	99,376	117,692	18.4	2.29
	Bt11	1927	7.48	1889	1964	3.95	4.71
	pat	1695	8.04	1696	1695	− 0.06	− 1.38

Absolute bias value of > 25% is shown in bold

^a^Average of two laboratories for each dPCR technique was used for bias calculation

**Table 5:** Calculation of GM% using microfluidic-based dPCR results

DNA sample	Target	Average GM%	Average GM% laboratory 3	Average GM% laboratory 2	% bias laboratory 3 to laboratory 2	% bias microfluidic-based dPCR to ddPCR^a^
Pea	tE9	n.a.	n.a.	n.a.	n.a.	n.a.
GT73	tE9	84.6^b^	89.2	80.2	11.3	10.6
GT73	GT73	57.5	59.6	55.4	7.87	1.90
MON88017	MON88017	53.9	53.5	54.3	− 1.46	1.23
Bt176	Bt176	4.26	3.96	4.51	− 12.2	2.99
Bt176	bar	4.21^b^	4.85	3.60	**34.9**	− 8.12
Bt11	Bt11	1.78	1.9	1.67	13.9	2.48
Bt11	pat	1.56^b^	1.71	1.44	18.5	− 3.54

Absolute bias value of > 25% is shown in bold

^a^Average of two laboratories for each dPCR technique was used for bias calculation

^b^The value is not actual GM%, because it was calculated based on the ratio between construct and endogene

When comparing the average absolute copy numbers produced by both laboratories for each event or gene, the overall cv was never higher than 25% (Table 4). However, when comparing the average copy number values between the laboratories, a problem with the bias slightly over 35% was again detected with Bt176 only. In addition, the data obtained with both types of dPCR were compared and the bias of microfluidic-based dPCR to ddPCR is presented in Table 4. Only in one case, for the tE9 gene in GT73, the bias was just above 25%. However, a tendency of producing higher absolute values with microfluidic-based dPCR can be observed, since the measured bias is in almost all cases in positive direction (Table 4).

Further, the GM% for the different materials was calculated. For most of the tested genes and events, the determined GM% values were in accordance between both laboratories with a bias below 25%. However, again a higher bias was observed for the bar target in Bt176 maize line (Table 5). It was also shown that there is no tendency for one laboratory to produce over- or under-estimated results, since the calculated bias was deviating in both directions (Table 5). The absolute bias between both types of dPCR in the case of measured GM% was not higher than 10% for any of the materials or genes/events and was not pointing in one direction as for the absolute copy numbers (Table 5).

### Inter-laboratory comparison of real-life sample quantification with ddPCR and qPCR

To see whether the quantification using ddPCR is comparable to the one with qPCR, two routine real-life samples, one containing two soybean transgenic events and another one containing three maize transgenic events, were tested on both platforms side-by-side. For the results to be directly comparable, the average GM% obtained by ddPCR (for maize events) was converted to mass fraction in two ways: (1) by taking into account the EURL technical guidance 619/2011 [5] and (2) by using the ratio measured with ddPCR (Table 3). The results of these quantifications are presented in Table 6. No difference (bias < 25%) can be seen between ddPCR and qPCR quantification of soybean events (as they are homozygous and there is no problem of parental origin). When comparing the bias of ddPCR to qPCR for maize events, using two different conversion factors, the absolute bias exceeded the threshold value of 25% only for DAS59122 in the case of using the actual conversion factor. Generally, when using the official conversion rate from the guidance document, we tend to under-estimate (for GMOs with male GM parent) or over-estimate (for GMOs with female GM parent) the GM content in comparison to the use of the actual conversion rate from the copy number determination. In most of the cases, this under-/over-estimation is still within acceptable limits. We have shown with our experiments that for a GM maize line, where the GM parent is male, there is a visible effect of the conversion from copy number ratio to mass fraction when using actual factors. In the same example (G154/13 DAS59122 in Table 6), the difference (bias) between both GM values (calculated with both approaches) is between 30 and 40%, what is already higher than the accepted 25% variation.

**Table 6:** Quantification of real-life samples with ddPCR and qPCR

Sample and event	GM% ddPCR	GM% qPCR laboratory 2	GM% qPCR laboratory 3	GM% qPCR average	Average GM% ddPCR (mass fraction)^a^	Average GM% ddPCR (mass fraction)^b^	% bias ddPCR to qPCR^c^	% bias ddPCR to qPCR^d^
G035/14 MON40-3-2	48.8	53.0	47.7	50.4	48.8	45.6	− 3.10	− 9.50
G035/14 MON89788	21.1	23.0	17.6	20.2	21.1	22.3	3.80	9.90
G154/13 DAS59122^e^	1.07	2.50	2.30	2.40	2.15	3.05	− 10.3	**27.2**
G154/13 DAS1507	2.28	4.50	3.38	3.94	4.55	3.93	15.5	− 0.20
G154/13 NK603	2.05	4.10	3.68	3.89	4.11	4.07	5.70	4.60

Absolute bias value of > 25% is shown in bold

^a^Calculated based on 0.5 conversion from EURL technical guidance 619/2011 [5] (not applicable for sample G035 as it contained homozygous soybean material)

^b^Calculated based on original ratio derived from ratio of calculated GM%/certified GM% (see Table 3)

^c^ddPCR value used in this calculation came from 0.5 conversion to mass fraction (for sample G035 non-converted GM% was used)

^d^ddPCR value used in this calculation came from original ratio conversion to mass fraction

^e^Male GM parent

### Calculated and measured copy numbers can be different

For the in-house validations of screening assays performed within the GMOval project, the samples were prepared to theoretically contain 5, 10, or 20 HGE of the respective targets. With dPCR, the absolute numbers of target copies in the samples of pea, GT73, MON88017, Bt176, and Bt11 were determined and compared to the theoretically calculated ones (Table 7). The theoretical values represent calculated HGEs (copies), which originated from the developer’s data [18] (calculations of HGE based on DNA concentration and dilution to the three presented levels).

**Table 7:** Calculated (in-house validation data) and determined copy numbers in samples used in method validation

Material	Gene/construct/event	HGE (copies)		
		Theoretical	Measured ddPCR	Measured microfluidic-based dPCR
MON88017	MON88017	20	15	18
		10	8	9
		5	4	4
GT73	GT73	20	28	38
		10	14	19
		5	7	10
	tE9	20	38	56
		10	19	28
		5	9	14
pea	lectin	20	48	61
		10	24	30
		5	12	15
	tE9	20	36	55
		10	18	28
		5	9	14
Bt11	Bt11	20	17	20
		10	9	10
		5	4	5
	pat	20	16	17
		10	8	9
		5	4	4
Bt176	Bt176	20	37	40
		10	18	20
		5	9	10
	bar	20	35	39
		10	18	20
		5	9	10

*HGE* haploid genome equivalents

For MON88017, the screening element was not measured, since the method for Cry3Bb used in the GMOval project was based on SYBR Green chemistry; therefore, only the copies of the event were determined. Results have shown that measured copy numbers of corresponding event, gene, or construct are similar to the calculated one only for MON88017 and Bt11 GM maize material (Table 7). For other materials, the measured values of corresponding event, gene, or construct were at least one and a half times higher (Table 7), indicating the presence of the screening element or the whole transgenic construct in more copies or just showing that the calculated HGE are not accurate.

In the case of Bt11 and Bt176, the copy number of constructs and screening elements was determined. As both materials were of the same GM%, the results (Table 7) are indicating that there are two Bt176 construct copies per HGE, compared to only one of the Bt11 construct. The same was seen for the respective screening elements bar (Bt176) and pat (Bt11). This result may be explained by the fact that, for Bt176, truncated versions of the transgenic insert are present [22], which might all (or only some of them, depending on the sequence) be detected, because of the use of the construct-specific method. For Bt11, both construct and screening element were determined to be present in equal ratio and the value was in accordance with the theoretical one.

## Discussion

Digital PCR is a technique that is gaining more and more attention, leading to an increased number of laboratories having the equipment available. The aim of our study was an inter-laboratory comparison of different dPCR platforms and their applicability for GMO testing. The experiments were performed on droplet and microfluidic-based digital PCR systems.

All the assays used in this work were developed and validated for qPCR. It was shown previously that individual assays can be transferred from qPCR to ddPCR [8–10]. To upgrade these studies, we decided to make an inter-laboratory comparison of direct transfer of several qPCR assays for detection/quantification of GMOs to the dPCR system without any optimization. We showed that the methods performed well in both dPCR systems, with coefficients of variation (inter-laboratory) and bias compared to qPCR), being within the acceptable limits [20]. However, we have observed that in some cases optimization of assays would be helpful to improve absolute quantification. The decision for optimization should thus be taken on a case by case basis.

Using a ddPCR system, the difference between two laboratories in terms of absolute copy numbers measured by individual assays easily exceeded the accepted 25% bias threshold [20] (Table 1). However, because the GM content is usually expressed in relative percent of GM per plant species (measured as ratio of GM event quantity versus the quantity of endogene) [23], this difference in absolute copies between laboratories does not interfere with the final percentage, as long as both assays for GM event and endogene behave in the same way. An interesting thing noticed in the case of ddPCR was the difference in number of analyzed droplets between the laboratories (results not shown), where values differed in some cases for almost two times. This of course does not affect the final calculated concentration, but it does point out differences in technical work (droplet generation, pipetting the droplets, etc.). Since the bias calculated for absolute copy number values for the same target was not always pointing in one direction, the reason for this between-laboratory variation most probably lies not in the methods themselves but rather in the samples, technical work or in the way of threshold determination during analysis of the results. This can be seen in the case of hmgA, where the assay is the same in all cases. The bias between laboratories for some samples is in one direction, for other cases in another, or there is no bias at all. Apart from technical work, another possibility for different absolute copy numbers could be different size (volume) of the droplets. It was already shown that the droplet size was not what was reported by the producer [13]; thus, this might be a plausible explanation.

When comparing the results of the microfluidic-based dPCR with ddPCR, a tendency to over-estimate (or under-estimate with ddPCR) the number of target copies was observed, but in most cases, this under- or over-estimation is still within an acceptable 25% variation range. This phenomenon was already observed at NIB in the past (unpublished data). The reporting of correction for actual size of droplets of 8% can reduce this difference [13]. However, on the level of GM% content, almost no differences were observed between both dPCR systems.

When using dPCR systems for GMO quantification, by measuring the absolute copy numbers, the results are consequently reported in copy number ratios. To be in line with regulations, the results should thus be converted to GM mass percentage. As far as the comparison to qPCR is concerned, dPCR produces similar results, when dPCR values are converted to mass fraction using the conversion factors established by our study. However, when comparing our results (Table 3) to previous ddPCR studies [8, 9], it can be clearly seen that there are huge differences in some batches of reference materials. Nevertheless, the discrepant cases could also be only an artifact due to incorrect reporting of the results in previous reports (e.g., wrong formula used for calculation of the factors). The conversion factors determined in our studies are generally in line with the ones already discussed by Holst-Jensen et al. [21] based on the ploidity of the GM parents. The problem of conversion between copy number ratio from dPCR measurements to mass fraction, for comparison to qPCR, is mainly in heterozygous (e.g., maize) samples, where there is an average twofold difference. The same problem could appear with analysis of multiploid species. The results presented in this manuscript can therefore be used as a starting point to determine all conversion factors, as recommended in the document prepared by EC JRC [7].

Additionally, as a comparison of inter-laboratory performance of the assays, we determined copy numbers in samples used for ring trial validations of screening assays for GMOval project [15]. In the process of in-house validations, the extracted gDNA is used to make a dilution series in order to determine the LOD (and eventually the PCR efficiency and linearity). The calculation of the number of copies of each dilution point is usually done by using the measured DNA concentration, the GM% of the material (certified GM% for CRM) and the nuclear DNA content as determined by Arumuganathan and Earle [6]. As the nuclear DNA content varies from study to study, this can be a source of error in the final copy number estimation.

It is easy to determine the absolute target copy number by dPCR (Table 7), but the values should probably not be directly compared to the ones calculated from the nuclear DNA content, because of possible multiple copies of the targeted PCR constructs. This could be nicely seen in the case of Bt11 and Bt176, where both materials were certified at 5% GM, but the absolute copy number of constructs was approximately two times higher in Bt176, indicating the presence of two copies of the Bt176 construct. The origin of the problem probably lies in a fact that the certification is not based on actual presence of DNA targets but rather on the mass fraction of GM seeds. The problem of differences in calculated and measured copy numbers was also noticed for the pea material, where the measured copy number was higher than the calculated ones for the lectin as well as for the tE9 sequence. It also seems that the values for GT73 event and the E9 terminator are not the same, meaning that more copies of tE9 could be present in GT73. In this view, the difference in results between the detection of the event using an event-specific qPCR method and the detection of a GM element using a screening method should be taken into account. Current transformation techniques enable introduction of different multiple unique inserts of the GM cassette (promoter-gene-terminator) in the host genome. Using a screening method, a specific element in this cassette is detected. When two or more inserts of the cassette are present, the element will be detected in each insert. The event-specific method, however, is directed towards the junction between the cassette and the plant genome. As this junction sequence is unique to a GMO, the event-specific qPCR method will detect only one cassette as only one will have the right junction sequence (the others having been integrated at other genomic loci and thus having other junction sequences).

The data obtained here are relevant in view of the determination of parameters, such as the LOD, of construct and element specific methods, which could be affected (e.g., higher or lower LOD). If the calculated copy number for an element is based on the GM% of the event and the element is present in more copies, the calculated copy number may be biased.

It was already shown by the reported data that the dPCR is suitable for the purpose of GMO detection by transfer of validated qPCR methods or even with new multiplex assays as already reported in other studies [24, 25]. Moreover, ddPCR is even more cost effective than qPCR, when performing the analysis of more samples together [26]. One of the reasons for this is the smaller number of reactions needed for quantification, as less dilutions of the samples can be used and there is no need for a calibration curve using CRMs.

In conclusion, our results indicate that due to simple transfer of assays from qPCR to dPCR, with no or few optimization steps needed, dPCR has a great potential to replace qPCR as the method of choice for DNA target quantification. However, one must choose the methods carefully and bear in mind the possibility of multiple copies of insert or endogene. Digital PCR, with its ability to measure the absolute number of target molecules, seems to be a great tool to determine the actual target copy number in a material or sample under investigation. We demonstrated that both types of dPCR are fit for this purpose. However, some care should be taken when comparing the results between laboratories. Additional investigations involving several laboratories may be needed to shed more light on the inter-laboratory variability.

## References

[1] Caprioara-Buda M, Meyer W, Jeynov B, Corbisier P, Trapmann S, Emons H (2012). Evaluation of plasmid and genomic DNA calibrants used for the quantification of genetically modified organisms. Anal Bioanal Chem.

[2] European Commission. Comission Regulation (EC) No. 641/2004 of 6 April 2004 on detailed rules for the implementation of Regulation (EC) No. 1829/2003 of the European Parliament and the Council as regards the application for the authorization of new genetically modified food. Off J Eur Union. 2004;14–25.

[3] AOCS Certified Reference Materials. 2017. https://www.aocs.org/crm. Accessed 7 Sep 2017.

[4] JRC Certified Reference Materials Catalogue. 2017. https://crm.jrc.ec.europa.eu/. Accessed 7 Sep 2017.

[5] European Commission. Comission Regulation (EU) No. 619/2011 of 24 June 2011 laying down the methods of sampling and analysis for the official control of feed as regards presence of genetically modified material for which an authorisation procedure is pending or the authorisati. Off J Eur Union. 2011;9–15.

[6] Arumuganathan K, Earle E (1991). Nuclear DNA content of some important plant species. Plant Mol Biol Report.

[7] Corbisier P, Barbante A, Berben G, Broothaerts W, De Loose M, Emons H, et al. Recommendation for the unit of measurement and the measuring system to report traceable and comparable results expressing GM content in accordance with EU legislation. Luxembourg; 2017. 10.2760/177516.

[8] Köppel R, Bucher T (2015). Rapid establishment of droplet digital PCR for quantitative GMO analysis. Eur Food Res Technol.

[9] Köppel R, Bucher T, Frei A, Waiblinger H-U (2015). Droplet digital PCR versus multiplex real-time PCR method for the detection and quantification of DNA from the four transgenic soy traits MON87769, MON87708, MON87705 and FG72, and lectin. Eur Food Res Technol.

[10] Gerdes L, Pecoraro S, Gerdes L, Iwobi A, Busch U, Pecoraro S (2016). Optimization of digital droplet polymerase chain reaction for quantification of genetically modified organisms. Biomol Detect Quantif.

[11] Morisset D, Štebih D, Milavec M, Gruden K, Žel J (2013). Quantitative analysis of food and feed samples with droplet digital PCR. PLoS One.

[12] Corbisier P, Bhat S, Partis L, Xie VRD, Emslie KR (2010). Absolute quantification of genetically modified MON810 maize (Zea mays L.) by digital polymerase chain reaction. Anal Bioanal Chem.

[13] Corbisier P, Pinheiro L, Mazoua S, Kortekaas AM, Chung PYJ, Gerganova T (2015). DNA copy number concentration measured by digital and droplet digital quantitative PCR using certified reference materials. Anal Bioanal Chem.

[14] Burns MJ, Burrell AM, Foy CA (2010). The applicability of digital PCR for the assessment of detection limits in GMO analysis. Eur Food Res Technol.

[15] GMOval Project. 2017. http://projects.nib.si/gmoval/. Accessed 7 Sep 2017.

[16] International Organization for Standardization (2005). ISO 21571:2005 foodstuffs—methods of analysis for the detection of genetically modified organisms and derived products—nucleic acid extraction. ISO 21571.

[17] GMO Methods. 2017. http://gmo-crl.jrc.ec.europa.eu/gmomethods/. Accessed 7 Sep 2017.

[18] Debode F, Huber I, Macarthur R, Rischitor PE, Mazzara M, Herau V (2017). Inter-laboratory studies for the validation of two singleplex (tE9 and pea lectin) and one duplex (pat/bar) real-time PCR methods for GMO detection. Food Control.

[19] Brodmann PD, Ilg EC, Berthoud H, Herrmann A (2002). Real-time quantitative polymerase chain reaction methods for four genetically modified maize varieties and maize DNA content in food. J AOAC Int.

[20] EURL-GMFF. Minimum performance requirements for analytical methods of GMO testing. 2015. http://gmo-crl.jrc.ec.europa.eu/doc/MPRReport Application 20_10_2015.pdf. Accessed 22 Feb 2016.

[21] Holst-Jensen A, De Loose M, Van den Eede G (2006). Coherence between legal requirements and approaches for detection of genetically modified organisms (GMOs) and their derived products. J Agric Food Chem.

[22] Moens W. Report on the molecular characterization of the genetic map of event Bt176. 2003. http://www.biosafety.be/gmcropff/EN/TP/MGC_reports/Report_Bt176.pdf. Accessed 28 Jul 2017.

[23] European Commission. Recommendation 787/2004 of 4 October 2004 on technical guidance for sampling and detection of genetically modified organisms and material produced from genetically modified organisms as or in products in the context of Regulation (EC) No. 1830/2003. Off J Eur Union L. 2004;18–26.

[24] Dobnik D, Štebih D, Blejec A, Morisset D, Žel J (2016). Multiplex quantification of four DNA targets in one reaction with Bio-Rad droplet digital PCR system for GMO detection. Sci Rep.

[25] Dobnik D, Spilsberg B, Bogožalec Košir A, Holst-Jensen A, Žel J (2015). Multiplex quantification of 12 European Union authorized genetically modified maize lines with droplet digital polymerase chain reaction. Anal Chem.

[26] Košir AB, Spilsberg B, Holst-Jensen A, Žel J, Dobnik D. Development and inter-laboratory assessment of droplet digital PCR assays for multiplex quantification of 15 genetically modified soybean lines. Scientific Reports. 2017;7:8601. 10.1038/s41598-017-09377-w. 10.1038/s41598-017-09377-w PMC5561262 28819142

